# Neuroregression, coarse features, and oligosaccharides in urines

**DOI:** 10.17712/nsj.2017.4.20170225

**Published:** 2017-10

**Authors:** Ahmed Y. BoAli, Kalthoum Tlili-Graiess, Amal M. AlHashem, Brahim M. Tabarki

**Affiliations:** *From the Divisions of Pediatric Neurology (BoAli, Tabarki), and Genetics (AlHashem), Department of Pediatrics, and the Division of Neuroradiology (Tlili), Department of Radiology, Prince Sultan Military Medical City, Riyadh, Kingdom of Saudi Arabia*

## Clinical Presentation

**Figure 1:**
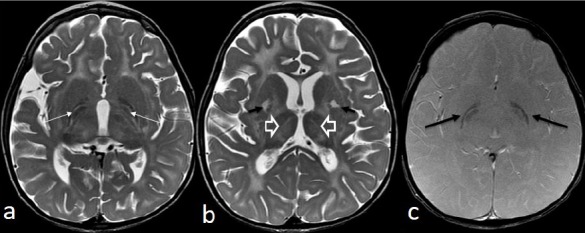
A brain MRI/MR axial **(a and b)** T2-weighted MR images show severe hypomyelination with a low signal of the globus pallidus within the medial and lateral segments separated by the hyperintense medial medullary laminae (white long arrows). The internal medullary laminae of the thalami are hyperintense (open arrows). A high signal intensity alteration is seen in both the putamina (short black arrows). The gradient echo weighted image **(c)** clearly shows the low signal of the globus pallidus (long black arrows)

**Figure 2:**
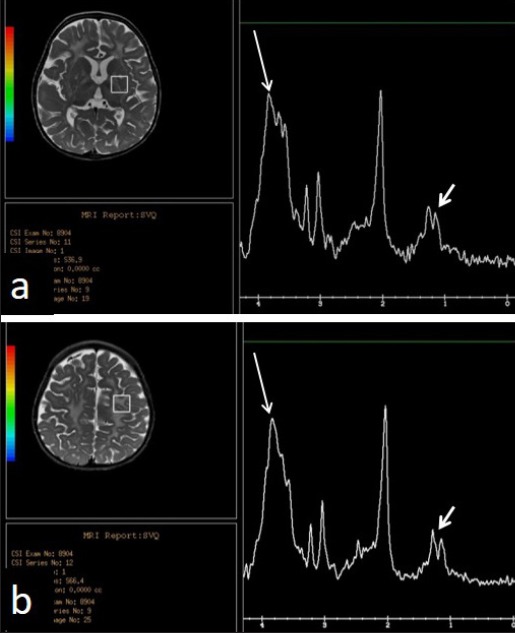
Single voxel MR spectroscopy (TE 35 ms) in the left basal ganglia **(a)** and the anterior centrum semiovale **(b)** demonstrates an abnormal broad and high peak at approximately 3.5-3.9 ppm composed of multiple peaks, the highest at 3.8 compatible with mannose (long arrow) and a second peak at 1.2 ppm (short arrow) representing fucose (this peak can easily be confused with the lactate peak, usually at 1.3 ppm).

A 5-year-old boy, the product of a consanguineous marriage, presented with neuroregression; and spasticity since the age of one year. There were no seizures. His sister, aged 3 years had similar features. Examination revealed impaired cognition, generalized spasticity, brisk deep tendon reflexes, and bilateral extensor plantar responses. The head circumference was normal, and there was mild facial coarsening. There were no organomegaly, or angiokeratomas, and the ophthalmological examination was unremarkable. A brain MRI/MR spectroscopy was performed and is shown in (**Figures 1 and 2**).

## Questions:

1. What are the findings inferred from **Figures 1 and 2**
2. What is the most likely diagnosis?
3. How is the diagnosis confirmed?

## Answers & Discussion

1. The brain MRI revealed abnormalities within the periventricular and subcortical white matter, the globus pallidus, and the putamina. The MRS showed a peak at the 3.8-3.9 ppm
2. Based on the clinical presentation and the neuroimaging findings, the most likely diagnosis is fucosidosis.
3. Confirmatory diagnosis of fucosidosis requires positive test for urine oligosaccharides, absent α-L-fucosidase activity in the plasma and leukocytes, and genetic study. In our patient, the urine was positive for oligosaccharides, and the genetic study showed a mutation in the FUCA1 gene (c.1123del). Fucosidosis is an autosomal recessive lysosomal storage disease caused by defective alpha -L- fucosidase, with accumulation of fucose in the tissues. Clinical features include progressive regression of milestones, spasticity, coarse facial features, angiokeratoma, and dysostosis multiplex.^1^-^3^ Fucosidosis has been classified into 2 major types. Type 1 is characterized by an early onset, rapid and severe psychomotor regression, and death within the first decade of life. Type 2 is characterized by milder neurological phenotype, the development of angiokeratoma, and longer survival.^1^-^2^ Neuroimaging plays an important role in the diagnosis of fucosidosis. The MRI of the brain shows prominent and progressive changes in white matter signal intensity, including periventricular, lobar, and subcortical supratentorial areas, internal and external capsules, internal medullary laminae of the thalami, putamina, and both hypothalami. The globi pallidi and substantia nigra have high signal intensity on T1 and low signal intensity on T2 and FLAIR. The MR sprectroscopy shows a broad and high “hump” corresponding to multiple peaks from approximately 3.5-3.9 ppm, presumed to be the result of accumulation of carbohydrate containing macromolecules. The highest peak at 3.8 is compatible with mannose and a doublet at 1.2 ppm, which invertsat long echotime, suggests fucose peak. The combination of imaging and MR spectroscopy findings is probably specific.^1^-^2^ Supportive diagnosis of fucosidosis also includes dysostosis multiplex; and high amounts of oligosaccharides in the urine.^1^-^3^ The treatment of fucosidosis is mainly supportive. Hematopoietic transplant or umbilical cord blood transplantation could reduce the severity and retard the progression of clinical neurological deterioration.^3^
